# A systematic review and meta-analysis of social cognition training success across the healthy lifespan

**DOI:** 10.1038/s41598-022-07420-z

**Published:** 2022-03-03

**Authors:** Mandy Roheger, Kseniya Hranovska, Andrew K. Martin, Marcus Meinzer

**Affiliations:** 1 Department of Neurology, University Medicine Greifswald, Walther Rathenau Str. 49, 17489 Greifswald, Germany; 2 Department of Psychology, University of Kent, Canterbury, CT2 7NP UK

**Keywords:** Neuroscience, Psychology

## Abstract

Socio-cognitive abilities and challenges change across the healthy lifespan and are essential for successful human interaction. Identifying effective socio-cognitive training approaches for healthy individuals may prevent development of mental or physical disease and reduced quality of life. A systematic search was conducted in MEDLINE Ovid, Web of Science Core Collection, CENTRAL, and PsycInfo databases. Studies that investigated different socio-cognitive trainings for healthy individuals across the human lifespan assessing effects on theory of mind, emotion recognition, perspective taking, and social decision making were included. A random-effects pairwise meta-analysis was conducted. Risk-of-Bias was assessed using the Cochrane Risk-of-Bias-2-Tool. Twenty-three intervention studies with N = 1835 participants were included in the systematic review; twelve randomized controlled trials in the meta-analysis (N = 875). Socio-cognitive trainings differed regarding duration and content in different age groups, with theory of mind being the domain most frequently trained. Results of the meta-analysis showed that trainings were highly effective for improving theory of mind in children aged 3–5 years (SMD = 2.51 (95%CI: 0.48–4.53)), children aged 7–9 years (SMD = 2.71 (95%CI: − 0.28 to 5.71)), and older adults (SMD = 5.90 (95%CI: 2.77–9.02). Theory of mind training was highly effective in all investigated age-groups for improving theory of mind, yet, more research on transfer effects to other socio-cognitive processes and further investigation of training effects in other socio-cognitive domains (e.g., emotion recognition, visual perspective taking, social decision making) is needed. Identified characteristics of successful socio-cognitive trainings in different age groups may help designing future training studies for other populations.

**Registration:** www.crd.york.ac.uk/PROSPERO/ (ID: CRD42020193297).

## Introduction

Social cognition is essential for successful human interaction and comprises processes relevant for understanding others’ emotions, perspectives, and mental states in order to interpret, explain, and predict the behavior of others^1^. The social requirements placed on humans are subject to considerable change across the lifespan. Moreover, cognitive processes that facilitate adaptation to novel and challenging social environments are subject to maturation and degeneration, similar to the patterns observed in other cognitive domains^1^.

For example, while development of socio-cognitive capacity starts in the first year of life^2^, significant changes in the ability to attribute mental states to other people (Theory of Mind, ToM) occur around the age of four, when children begin to more accurately interpret emotions and intentions of others^3^. ToM is a multidimensional construct that can be differentiated into two subcomponents: cognitive ToM, which describes a cognitive understanding of the difference between the speaker’s knowledge and that of the listener, and affective ToM, which describes the empathic appreciation of the observed person’s emotional state^4^. ToM is necessary in order to understand and predict the behavior of others^5^ and is a prerequisite for successful social interaction^6^. However, there is substantial variability in ToM development and other socio-cognitive skills and different mechanisms may underlie the diverse aspects of social cognition^7^. Neuroanatomical correlates involve for example the medial prefrontal cortex structures, the superior temporal sulcus region, the temporal poles, and the amygdala^8^. This inter-individual variability is crucially shaped by a range of genetic and environmental factors^9,10^. Therefore, development and adaptation of socio-cognitive skills across early life is an active process and strongly influenced or *trained* by the environment children are exposed to^11,12^. Consequently, there is a need to extend the contexts in which ToM and other socio-cognitive skills are investigated to include settings such as kindergarten and pre- and middle schools^13^ to properly account for social factors and interactions in children’s development^14^. This is important as ToM abilities continue to develop into adulthood (e.g.^15^).

Throughout adolescence and adulthood, humans face considerable social challenges associated with puberty, the end of the schooling period and entering life as an independent adult^16^. Social demands continue to change throughout adulthood and towards the third age. Advanced age is also associated with cognitive decline, which has marked effects on social functioning^1^. For example, there is evidence that healthy older adults may have difficulties in inferring mental states in complex social scenarios^17^ and even basic emotion recognition may decline (for review see Arioli et al. 2018). Furthermore, decline of ToM in older age can occur due to changes in brain structure and function: For example, a recent study showed that older adults exhibit weaker intrinsic connectivity between the right temporoparietal junction and right temporal pole that explained their poorer ToM behavioral performance^18^.

Importantly, impairment of social functioning in later life has been linked to mental^19^ and physical problems^20^, functional disability^21^, and reduced quality of life^22^. Moreover, socio-cognitive impairment is a core feature of many neurodegenerative disorders such as frontotemporal dementia or Alzheimer’s disease^23^ and also an early and salient marker of many neurodevelopmental, neuropsychiatric disorders^23,24^. Consequently, there is considerable research interest in designing socio-cognitive training approaches that foster socio-cognitive development or prevent age-related decline across the human lifespan.

Despite the importance of social cognition for successful development and aging, no previous study has provided an overview of the different socio-cognitive training approaches currently being used across the healthy human lifespan. This will be addressed in the present review that aims at (a) systematically describing all socio-cognitive trainings for healthy individuals over the lifespan and (b) investigating the effect of socio-cognitive trainings in healthy individuals at different ages using a meta-analytical approach.

## Results

### Results of the search

**Figure 1:**
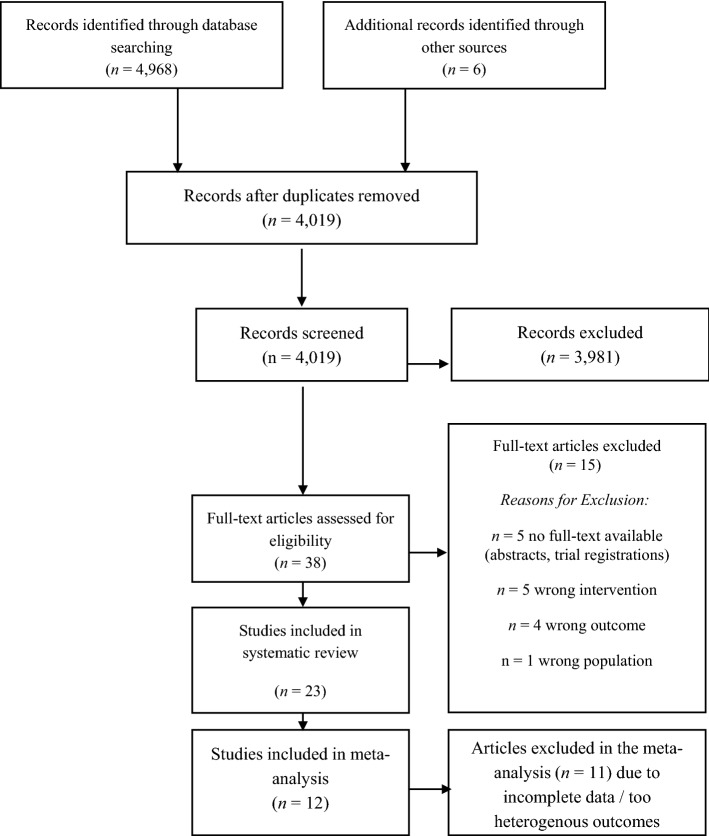
PRISMA diagram illustrating the study selection process.

The initial search of databases yielded *n* = 4968 studies and an additional *n* = 6 studies were identified through scanning of relevant reviews. After removal of duplicates *n* = 4019 studies were screened. After abstract and title screening, we assessed 38 full-texts for eligibility and included 23 studies in the systematic review. *N* = 12 studies were included in the pairwise meta-analysis. The PRISMA flow-diagram^25^ in Fig. 1 provides an overview of the study selection process.

### Characteristics of socio-cognitive trainings

Twenty-three studies (21 RCTs, two quasi-experimental studies) with a total of 1835 participants investigating the effectiveness of socio-cognitive trainings were included. A detailed overview of included studies with information on participants (e.g., group sizes, age, sex), interventions, and investigated outcomes is provided in Table 1. The specific tests for outcome assessments and details of the results of each study are displayed in Table 2.

**Table 1:** Characteristics of included studies.

Study	Design	Participants			Intervention		Outcome				
Author, year and country		n	Age M (SD)	Sex	Name	Description (training content, overall number of sessions, training length per session, delivery mode )	Theory of mind	Emotion recognition	Social decision-making	Perspective taking	Others
**Age group: 0–18 years, children and adolescents**											
Bianco et al. (2019) Italy	Cluster-RCT	Group 1: 27 Group 2: 22	Overall: 7.60 years (3.98 months)	Group 1: 59.25%♀ Group 2: 68.18♀	Group 1: advanced theory of mind Group 2: control condition	Group 1: two misunderstandings, two sarcasms, two faux-pas und two double-bluff stories in increasing complexity Group 2: narratives and language exercises referred not to mental, but to physical states 4 sessions, twice a week, 50 min each Face to face group setting	x				Verbal ability, working memory, interference control, shifting, reading comprehension, metacognition
Bianco et al. (2021) Italy	Cluster-RCT	Group 1: 28 Group 2: 36 Group 3: 27	Overall: 7.59 years (3.97 months)	Group 1: 53.57%♀ Group 2: 50.00%♀ Group 3: 59.26%♀	Group 1: second-order reaction time Group 2: advanced theory of mind Group 3: control condition	Group 1: group conversations about narratives & two language exercises Group 2: two misunderstandings, two sarcasms, two faux-pas und two double-bluff stories in increasing complexity Group 3: narratives and language exercises referred not to mental, but to physical states 4 sessions, twice a week, 50 min each Face to face group setting	x				Verbal ability, working memory, interference control, shifting, reading comprehension, metacognition
Caputi et al. (2021) Italy	RCT	Overall: 210	Overall: 9.66 years (0.85)	Overall:48.00%♀	Group 1: theory of mind training Group 2: no-theory of mind training	Group 1: group discussion about mentalistic stories which were similar to target strange stories Group 2: group discussion about physical stories which were similar to target strange stories 5 weekly sessions, 50 min each Face to face group setting	x				Loneliness, verbal abilities, socio-economic status
Carbonero Martin et al. (2013) Spain	Quasi-experimental	Group 1: 10 Group 2: 10	n.a	Group 1: 50.0% ♀ Group 2: 50.0% ♀	Group 1: mentalist skills Group 2: control group	Group 1: metacognitive intervention program in which children learn to talk about other people’s mental states, weekly 45 min sessions for 3 months Group 2: no treatment Face to face group setting	x				Mentalist skills
Guajardo and Watson et al. (2002) USA	RCT	Group 1: 19 Group 2: 18	Overall: 46.0 (n.a.)^a^	Group 1: 45.0% ♀ Group 2: 33.3% ♀	Group 1: storytelling training Group 2: control group	Group 1: stories about maxi and his mother in different everyday situations with false-belief tasks in 12–15 sessions each lasting 15–25 min over a period of 5 weeks Group 2: no treatment Face to face group setting	x				Language
Hale and Tager-Flusberg (2003) USA	RCT	Group 1: 20 Group 2: 20 Group 3: 20	Group 1: 47.0 (5.1)^a^ Group 2: 48.4 (5.9)^a^ Group 3: 45.6 (6.3)^a^	Overall: 41.6% ♀	Group 1: false belief Group 2: sentential complements Group 3: relative clauses	Group 1: location change story Group 2: story about action towards a Sesame street character and questions about it Group 3: two twin characters performed tasks, children had to say which character did what Two training sessions Face to face group setting	x				Sentential complements, relative clauses
Lecce et al. (2014) Italy	RCT	Group 1: 33 Group 2: 29	Overall: between 4 and 5 years	Group 1: 51.5% ♀ Group 2: 34.4% ♀	Group 1: theory of mind training Group 2: control-physical condition	Group 1: ToM training focused on first-order false-belief tasks Group 2: participants practiced on control-physical stories (stories about events in the physical domains e.g. logical consequences involving humans and animals) Both training consisted of three 20 min sessions Face to face individual setting	x				Metamemory
Lu et al. (2008) China	RCT	Group 1: 26 Group 2: 25	Group 1: 43.9 (4.4)^a^ Group 2: 43.9 (4.9)^a^	Group 1: 46.1% ♀ Group 2: 56.0% ♀	Group 1: theory of mind training Group 2: control training	Group 1: storytelling with questions regarding characters of the story Group 2: storytelling with questions regarding physical features Four sessions, 10–15 min each Face to face individual setting	x				
Ornaghi et al. (2021) Italy	RCT	Overall: 70	Overall: 3.10 years (5.96 months)	Group 1: 50.00% ♀ Group 2: 50.00% ♀	Group 1: theory of mind training Group 2: control training	Group 1: storytelling enriched with metal state language and language games Group 2: storytelling, but free play afterwards 2-month intervention, twice weekly sessions, 20 min each Face to face group setting	x				Metacognition, language, Pragmatic competence
Peskin et al. (2004) Canada	RCT	Overall: 48	Group 1: 4.5 (n.a.) Group 2: 4.7 (n.a.)	Group 1: 41.66%♀ Group 2: 45.83%♀	Group 1: theory of mind training Group 2: control training	Group 1: books with test rich in explicit metacognitive terms Group 2: same books without metacognitive language 4-week intervention Face to face group setting	x				Language, metacognition
Qu et al. (2015) Singapore	RCT	Overall: 71	Group 1: 59.4 (5.4)^a^ Group 2: 60.6 (5.6)^a^ Group 3: 60.2(6.1)^a^	Overall: 47.8%	Group 1: free play Group 2: sociodramatic play Group 3: sociodramatic play and theory of mind coaching	Group 1: books and several toys were provided for free play Group 2: ToM story, participants pretended to be in the story Group 3: similar to Group 2, with additional support Four weekly 45 min sessions Face to face group setting	x				Language, executive functions
Rostan et al. (2014) Spain	RCT	Overall: 78	Group 1: 43.8 (1.7)^a^ Group 2: 42.9 (1.6)^a^ Group 3: 43.2 (1.9)^a^	Group 1: 65.0% ♀ Group 2: 58.0% ♀ Group 3: 58.0% ♀	Group 1: SDO training Group 2: SDN training Group 3: LAB training	Group 1: sentential complements with deceptive objects (e.g. a candle in the shape of a tomato, children have to talk about what the object is) Group 2: sentential complements with non-deceptive objects Group 3: labelling of objects according to characteristics 3 training sessions, each 5–10 min Face to face group setting	x				Vocabulary
Serrat Sellabona et al. (2013) Spain	RCT	Overall: 104, 26 per group	Overall: 3.70 (n.a.)	n.a	Group 1: discourse training (DIS) Group 2: labelling training (LAB) Group 3: sentential complements with non-deceptive objects (SDN) Group 4: control group (CON)	Group 1: children needed to talk with a puppet about deceptive objects they saw Group 2: labelling of objects according to characteristics Group 3: sentential complements with deceptive objects (e.g. a candle in the shape of a tomato, children have to talk about what the object is) Group 4: deceptive objects were shown but nobody talked Three training sessions, 10 min each Face to face group setting	x				
**Age group: 18–60 years: young and middle aged adults**											
Alkozei et al. (2018) USA	RCT	Group 1: 31 Group 2: 31	Group 1: 27.1 (6.7) Group 2: 26.8 (8.1)	Group 1: 58.6% ♀ Group 2: 50.0% ♀	Group 1: internal awareness training Group 2: external awareness training	Group 1: program focused on understanding, perceiving, managing and using emotions Group 2: program focused on learning about external environment (e.g. plants). Both programs consisted of 6 lessons, twice a week for 3 weeks, each session lasting 30–45 min Online individual setting			x		Emotional intelligence
Haut et al. (2019) USA	RCT	Group 1: 24 Group 2: 21	Group 1: 24.5 (2.9) Group 2: 24.6 (2.9)	Group 1: 41.7% ♀ Group 2: 53.0% ♀	Group 1: social cognitive training Group 2: computer game control	Group 1: training focused on training facial emotion recognition, emotional prosody, perspective-taking Group 2: participants completed common computer games Both trainings consisted of 15 sessions, each 45 min Online individual setting				x	Empathy, intrinsic motivation
Kemney et al. (2012) USA	RCT	Group 1: 41 Group 2: 41	Overall: 41.1 (10.4)	Group 1: 100% ♀ Group 2: 100% ♀	Group 1: meditation/emotion training Group 2: waitlist control group	Group 1: concentration training, mindfulness, promotion of empathy, yoga, emotion recognition Group 2: waitlist control Training lasting 8 weeks (42 h) Face to face group setting		x			Mood, stress
Meyer et al. (2016) USA	RCT	Group 1: 27 Group 2: 27	Group 1: 21.4 (3.5) Group 2: 21.1 (2.1)	Group 1: 51.8% ♀ Group 2: 51.8% ♀	Group 1: social working memory training Group 2: cognitive working memory training	Group 1: ranking of friends in working memory training Group 2: alphabetical ranking in working memory training Both training consisted of twelve 20 min sessions Online individual setting				x	Working memory
Santiesteban et al. (2012) UK	RCT	Group 1: 19 Group 2: 17 Group 3: 17	Overall: 26.7(6.6)	n.a.	Group 1: imitation training Group 2: imitation-inhibition training Group 3: inhibitory control training	Group 1: training focuses on imitation of videos in which either an index or middle finger performed a lifting movement Group 2: training focuses on not imitating moves from a video, but rather do the opposite (lift the middle finger when the video shows the index finger) Group 3: stroop-task training Two training sessions, 40 min each Face to face individual setting	x			x	
Valk et al. (2017) Germany	RCT	Group 1: 80 Group 2: 81 Group 3: 81	Overall: 40.7(9.2)	Overall: 59.3% ♀	Group 1 and 2: affect, presence and perspective module Group 3: only affect module	Group 1 and 2 attended all three modules in a different order. Group 3 only attended the affect training. Trainings lasted 39 weeks, divided in 3 modules, each lasting 12 weeks Face to face group setting	x				Compassion, selective attention
**Age group: older than 60 years: older adults**											
Cavallini et al. (2015) Italy	RCT	Group 1: 37 Group 2: 26	Group 1: 71.4 (5.1) Group 2: 71.5 (5.6)	Group 1: 86.5% ♀ Group 2: 85.5% ♀	Group 1: theory of mind training Group 2: physical-conversation training	Group 1: ToM training focused on tasks and conversations about mental states Group 2: participants practiced and discussed material about physical occurrences Both trainings consisted of 4 sessions. No information on training frequency Face to face group setting	x				
Lecce et al. (2015) Italy	RCT	Group 1: 24 Group 2: 24 Group 3: 24	Group 1: 69.6 (7.3) Group 2: 65.5 (5.3) Group 3: 67.7 (5.9)	Group 1: 79.2% ♀ Group 2: 79.2% ♀ Group 3: 70.8% ♀	Group 1: theory of mind training Group 2: physical conversion training Group 3: social contact group	Group 1: ToM training focused on tasks and conversations about mental states Group 2: participants practiced and discussed material about physical occurrences Group 3: group conversations Two weekly 2-h training sessions Face to face group setting	x				Metamemory
Lecce et al. (2019) Italy	Quasi-experiment	Group 1: 43	Group 1: 68.3 (6.4)	Group 1: 66.6% ♀	Group 1: theory of mind training	Group 1: ToM training focused on tasks and conversations about mental states Three weekly 2-h training sessions Face to face group setting	x				Updating, set-shifting, verbal knowledge
Rosi et al. (2016) Italy	RCT	Group 1: 85 Group 2: 83	Group 1: 70.5 (6.9) Group 2: 68.4 (6.1)	n.a.	Group 1: theory of mind training Group 2: control group	Group 1: ToM training focused on tasks and conversations about mental states Group 2: participants practiced and discussed material about physical occurrences Four 2-h sessions. No information on training frequency Face to face group setting	x				Animation task

*RCT* randomized controlled trial, *ToM* theory of mind.

^a^Age of children was displayed in months.

**Table 2:** Results of the included studies.

Study	Outcome Assessment	Theory of Mind	Emotion Recognition	Social decision-making	Perspective Taking	Other	Follow-up
**Age group: 0–18 years, children and adolescents**							
Bianco et al. (2019)	Theory of mind (strange stories)	↑*					/
Bianco et al. (2021)	Theory of mind (strange stories)	↑*					/
Caputi et al. (2021)	Theory of mind (strange stories)	↑*				Subgroup analysis for gender	/
Carbonero Martin et al. (2013)	Theory of mind task (false belief)	↑*					/
	Mentalist skills register					↑*	/
Guajardo and Watson et al. (2002), study 1	Composite theory of mind score (unexpected change task, unexpected content task, deception task, perceptual appearance-reality distinction tasks)	x					4–5 w
	Auditory comprehension of language-revised test					n.r	4–5 w
Hale and Tager-Flusberg (2003)	False belief test	↑* (both groups: false belief & sentential complement)					/
	Sentential complement test					↑* (only sentential complement group)	/
	Relative clause test					↑* (only relative clause group)	/
Lecce et al. (2014)	Theory of mind (two second-order false belief tasks, two belief–desire–reasoning tasks, and a selection of the theory-of-mind test components)	↑*					2 m
	Metamemory (metamemory vignette task)					↑*	2 m
Lu et al. (2008), study 2	Composite theory of mind score (four false belief tasks, two deception tasks)	↑*					/
Ornaghi et al. (2021)	False-belief understanding	↑*					/
	Emotion comprehension	↑*					/
Peskin et al. (2004)	False-belief explanation battery	↑*					/
	False-belief prediction battery	x					/
Qu et al. (2015)	Composite theory of mind score (false belief, location false belief, belief emotion)	Prediction analysis					/
	Language (PPVT-IV)					Prediction analysis	/
	Executive function composite (forward digit, backward digit, flexible item selection task)					Prediction analysis	/
Rostan et al. (2014)	Unexpected content task	↑					1.5 m
	Change of location task	↑					1.5 m
	Appearance-reality task	↑*					1.5 m
Serrat et al. (2013)	Unexpected content task	↑					/
	Change of location task	↑ (in DIS and LAB)					/
	Appearance-reality task	↑*					/
**Age group: 18–60 years: young and middle aged adults**							
Alkozei et al. (2018)	Iowa gambling task			x			/
	Emotional intelligence composite (bar-on emotional quotient inventory, The Mayer–Salovey–Caruso emotional intelligence test)					↑*	/
Haut et al. (2019)	Empathic accuracy task				↑*		/
	Intrinsic motivation inventory					Prediction analysis	/
Kemney et al. (2012)	Recognizing microexpressions of emotion on the face		↑*				5 m
Meyer et al. (2016)	Perspective-taking task				↑*		/
	Working memory task					↑*	/
Santiesteban et al. (2012)	Imitation-inhibition task	↑*					/
	Strange story task	x					/
	Director task	x					/
Valk et al. (2017)	Theory of mind	↑					/
**Age group: older than 60 years: older adults**							
Cavallini et al. (2015)	Theory of mind stories	↑*					/
	Theory of mind animations	x					/
Lecce et al. (2015)	Strange story task	x (compared to passive control)					
	Metarepresentational verbs task	↑* (compared to passive control)					/
	Metamemory questionnaire					↑* (compared to passive control)	/
Lecce et al. (2019)	Composite of strange story task and metarepresentational verbs task	↑*					/
	Composite score of vocal test on mental states and animation task	↑*					/
Rosi et al. (2016)	Strange story task	↑*					/
	Animation task	↑*					/

The table shows the results of the direct post-test assessment of the studies.

Follow-up results were not reported as follow-up lengths were too heterogeneous and not all studies conducted follow-ups.

↑ = experimental group performed better than control group. ↓ = experimental group performed worse than control group. * = significant results. X = no difference between experimental and control group. n.r. = not reported. w = weeks. m = months.

As described, we had initially defined three broad age brackets to cluster different ages ranges (0–18 years: children and adolescents, 18–60 years: young and middle-aged adults, older than 60 years: older adults). Yet, we acknowledge that these age brackets are broadly defined, e.g. substantial changes in socio-cognitive domains are observed between early life and the late teenage years^26^ and also in individuals aged 60 years and older^1^. Moreover, there are enormous differences in social challenges individuals face across development, young adulthood, middle and late age. Therefore, we further sub-divided the systematic review and meta-analysis where applicable.

#### Children and adolescents

In the lowest age bracket, thirteen studies investigated socio-cognitive training in children^27–39^. Eight studies were conducted in Europe^28,30,33–37,39^, two studies in The United States of America^27,29^, one in Canada^38^, and two studies in Asia^31,32^. The mean age of the investigated populations in this age range was 5.98 years (range 43–60 months). *N* = 10 studies reported training effects in kindergarten age (range:43–60 months); *n* = 3 studies included older children attending elementary school (range 7–9 years)^34,35,39^. Socio-cognitive training in both groups was labelled as either ToM-training, storytelling, or metacognitive intervention with the overall aim for children to further develop socio-cognitive skills using tasks where they had to reflect on other person’s mental states and beliefs. Training sessions for children in Kindergarten age were relatively short [between 15^27,30,31^ and 45 min^28^] and lead by a trained experimenter. There was substantial variability in training frequency and duration. Two to four training sessions per week were conducted^29–33^ over five^27^ to 20 weeks^28^. Training for children attending elementary school lasted 40–50 min to resemble the duration of a standard school lesson, and training was conducted up to five times per week^34,35,39^. Kindergarten age children participated in the training either individually^33^ or in small groups of up to five children^27–32^, children in elementary school attended the training in small groups. Stories used for ToM training in children typically introduced the protagonists first (e.g., animals or children with pleasantly sounding and easy to remember names like Monty and Freddy), followed by a simple story about them. Contents and questions asked by the experimenter varied depending on the goal of the training (e.g., emotion contingencies, false belief tasks). For example, in studies that targeted emotion contingency^30^, children were told that Monty likes one thing (e.g. apples) but dislikes another (e.g. pears). Monty then puts an apple in a box, leaves and Freddy joins the scene replacing the apple with a pear. Children would then be asked questions about “How Monty feels when he gets an apple”, “What Monty thinks about the box content when he returns” or “How he would feel when he opens the box”. Age-appropriate materials were used in these stories including cartoon pictures^32^, a storybook with illustrations to engage children^27,29–31^ or puppets^30^ and the trainers encouraged children to ask and answer questions about the stories and provided feedback. In children in elementary school aged between 7 and 9 years different ToM narratives were told, which were not supported by puppets or picture books, but rather discussed in a group setting with other children and teachers^34,35,39^. Overall, trainings did not focus on specific developmental challenges posed by kindergarten/school, but rather on basic socio-cognitive processes and skills.

#### Young and middle-aged adults

Six studies investigated effects of socio-cognitive training in young and middle aged adults [middle age bracket: mean age in the investigated studies ranged from 21.10 to 41.10 years^40–45^]. Four studies were conducted in The United States of America^40–43^, two studies in Europe^44,45^. Four of them used online or computerized socio-cognitive training^40,41,43,45^, focusing on one^43^ or several socio-cognitive tasks^40,41,45^ that were trained repeatedly. Other trainings were conducted in an offline group setting^42,44,45^. Duration of training sessions for young and middle aged adults ranged from 20^43^ to 45 min^40,41^. There was substantial variation in training frequency, yet, training in this age bracket was delivered at a higher frequency than in children and ranged from six^40^ to 42 sessions^42^. The only exception was the study conducted by Santiesteban and colleagues, who conducted only two training sessions^44^. Unlike in children, where trainings focused mainly on development of rather basic socio-cognitive processes and skills using simple tasks, there was no clear pattern of specific ToM-tasks used in the different trainings in young and middle-aged adults. Several more complex socio-cognitive tasks were trained including emotion recognition by the Reading the Mind in the Eyes task (in which participants can only see the eyes of a person and have to decide which emotion the eyes express out of a list of four emotions^41^) or a modified version of the Iowa Gambling Task, which practices social decision making^40^. The specific tasks were trained in an adaptive manner depending on the participant’s performance. Three of the studies used additional didactic presentations on socio-cognitive processes and strategies to enhance socio-cognitive abilities^40,42,45^, to provide theoretical background, and to introduce strategies to adapt to new situations. One study^43^ also trained working memory in addition to socio-cognitive tasks. Both studies that investigated ToM demonstrated significant improvement after the training (either on an broad ToM task^45^ or by using an Imitation-Inhibition Task^44^, in which participants were either asked to imitate a specific behaviour or inhibit its imitation). Three studies investigated emotion recognition^42^ or perspective-taking as primary outcomes^41,43^ and reported significant improvements after the end of the training period. No beneficial training effects were demonstrated in a study that used social decision making ability as primary outcome^40^. For additional information on these results see Table 2.

#### Older adults

In the upper age bracket, four studies investigated socio-cognitive training in adults older than 60 years [mean age range between 68.30 and 71.50 years^46–49^]. All four studies were conducted by the same Italian group and used similar socio-cognitive training approaches, designed as RCTs and one quasi-experimental design without a control group^48^. All ToM trainings focused on tasks and extended conversations about mental states. ToM tasks were practised in small groups with experienced experimenters as group leaders, who provided feedback on task performance. Specifically, a trainer presented one or two written ToM stories in each session and then asked several questions about the main character’s mental state, one character’s belief about the other character’s mental state, mental states underlying specific social behaviour and what the main character could do or say in order to change the other character’s mental state. ToM stories varied in content and complexity including misunderstandings, double bluffs, or sarcasm. Answers were then discussed in a group setting. Two^47,48^ to four sessions were completed^46,49^, with each session lasting up to two hours. Two studies reported that the training sessions were conducted weekly^47,48^, whereas two studies did not provide information on training frequency^46,49^.

### Results of the meta-analysis

**Figure 2:**
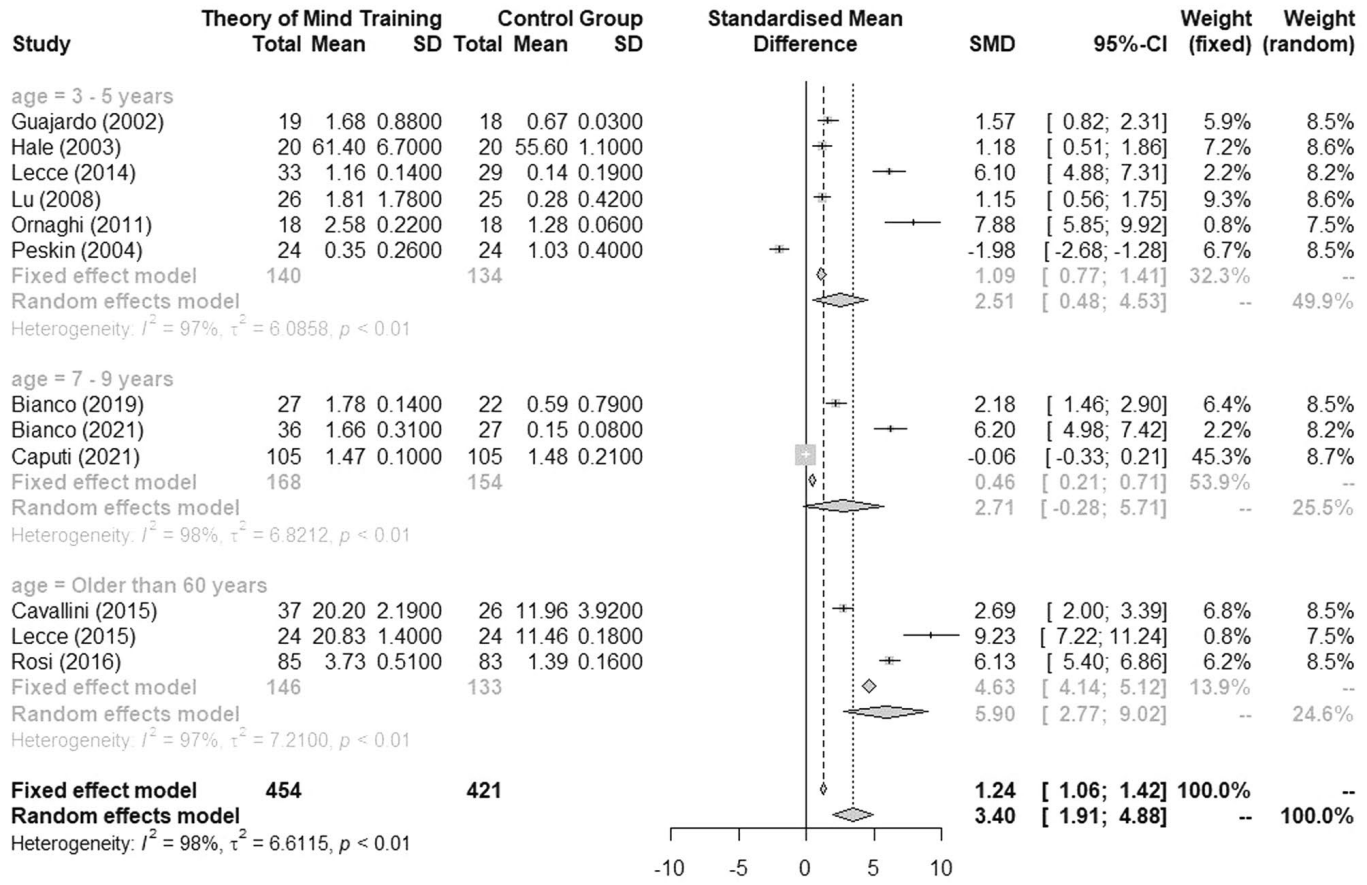
Forest plot of meta-analysis on the effectiveness of theory of mind training compared to control group, arranged by age groups.

Twelve RCTs provided data on our primary outcome (ToM) and were included in a random-effects pairwise meta-analysis. The forest plot of the main analysis is displayed in Fig. 2. Based on the outcome of the systematic review, we post-hoc decided to further cluster our analysis for the lowest age bracket (0–18 years) in two groups: children between 3 and 5 years and children aged between 7 and 9 years. In total, 875 participants were included in this analysis (n = 140 children aged between 3 and 5 years in the ToM training; n = 134 children aged between 3 and 5 years in the control group; n = 168 children aged between 7 and 9 years in the ToM training; n = 154 children aged between 7 and 9 years in the control groups; n = 146 adults older than 60 years in the ToM training; and 133 adults older than 60 years in the control group). Studies eligible from the middle age bracket did not provide sufficient data to justify inclusion into the meta-analysis, as a minimum of n ≥ 2 studies with comparable data is needed to calculate a meta-analysis (Deeks et al., 2021).

The overall effect size, the standardized mean difference (SMD), was 3.40 (95%CI: 1.19–4.88), indicating a large effect size, which was statistically significant (p < 0.01). However, I^2^ was high (98%), pointing to substantial heterogeneity between studies. Subgroup analysis also showed a large effect size (SMD = 2.51 (95%CI: 0.48–4.53)) for children aged between 3 and 5 years and an even larger effect size for children aged between 7 and 9 years (SMD = 2.71 (95%CI: − 0.28 to 5.71)) . We also identified a strong training effect for adults older than 60 years (SMD = 5.90 (95%CI: 2.77–9.02)). However, there was substantial heterogeneity in all comparisons (children aged 3–5 years: I^2^ = 97%, children aged 7—9 years: I^2^ = 98%, adults older than 60 years: I^2^ = 97%).

To provide additional confirmatory information for these results, additional sensitivity analyses with fixed-effects models were conducted and results are displayed in Fig. 2, showing lower effect sizes than the random-effects model (overall SMD = 1.24 (95%CI: 1.06– 1.42)). A sensitivity analysis dividing the studies according to different theory of mind tests used (e.g. false-belief task, strange story test, socio-cognitive composite score) are reported in the Supplementary Materials, Fig. 1. However, these sensitivity analyses did not reduce the heterogeneity of the studies and could only be conducted for children (including all studies, regardless of age-clusters within in the group) because not enough data was available for the other comparisons (when considering three studies as minimum per subgroup). A funnel plot that was conducted over all studies further showed an uneven distribution, indicating a possible publication bias (see Supplementary Material, Fig. 2) for studies with higher effect sizes. Yet, it should be noted that we could only include a small number of studies and the funnel plot does not consider the quality of included studies and should therefore be interpreted with caution.

### Risk of bias analysis

The results of the risk of bias assessment are summarized in Table 3. Overall, most studies were rated as “with some concerns”, mainly because studies and/or analyses were not pre-registered and the randomization process and blinding was not clearly described. Two studies^28,48^ were rated as high risk of bias because they were not properly randomized, but quasi-experimental studies.

**Table 3:**
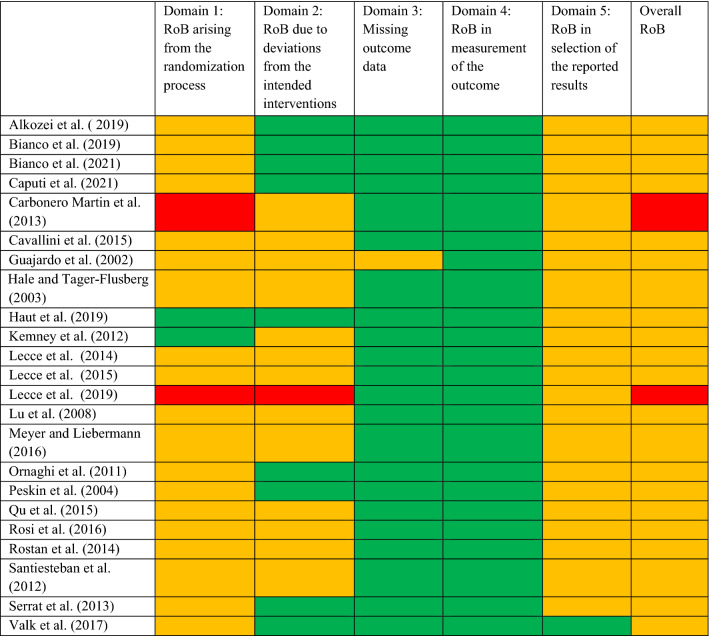
Risk of Bias (RoB) assessment of included studies.

Red color indicates a high risk of bias, yellow color indicates a medium risk of bias, green color indicates a low risk of bias, assessed with the Revised Cochrane risk-of-bias tool for randomized trials (RoB 2).

*RoB* risk of bias.

## Discussion

This is the first systematic review and meta-analysis of socio-cognitive training approaches that are currently used in healthy individuals across the human lifespan. Results of the 23 studies included in the systematic review showed that socio-cognitive trainings differed across age groups with regard to their settings, methods, frequency, duration, and training elements. Notably, 10 of the 23 included studies focused on very young healthy children around four years of age. Results of the meta-analysis for studies that specifically targeted ToM outcomes and included twelve studies with 875 participants demonstrated that (a) socio-cognitive trainings yielded large effect sizes and, (b) effects were most pronounced in healthy older adults (> 60 years of age). The middle age bracket could not be included into the meta-analysis, because there was not enough data available for the different outcomes. However, five of the six studies included in the systematic review also reported positive effects of socio-cognitive training targeting ToM^44,45^, emotion recognition^42^, and perspective-taking^41,43^. Risk of bias assessment revealed some methodological concerns in all included studies, mostly because of failure to pre-register studies or missing information on the randomization process.

Different stages of life pose unique challenges to social interaction and socio-cognitive abilities: as children, we start to develop the abilities to understand and communicate with others’, during puberty, adolescence and adulthood, we are constantly confronted with life changes that challenge our socio-cognitive skills and our ability to adapt these to new circumstances^16^. Finally, as we age, (socio-) cognitive abilities may start to decrease leading so social difficulties and reduced quality of life^17^. Therefore, socio-cognitive training may be helpful at every stage of life to either develop (new) socio-cognitive abilities, adapt our behaviour to new environments and challenges or to maintain a high-level of socio-cognitive functioning. Consequently, an important precondition for designing suitable training approaches for individuals of different ages is knowledge about specific challenges posed by different stages of life^47^, as well as adapting the training to individual needs. Training for younger children typically aimed at developing certain socio-cognitive skills and therefore focused explicitly on examples of every-day situations relevant for such young age in rather short training sessions (i.e., 5–20 min per session) in which ToM-stories were told by an experienced group leader, frequently an adult with a pedagogical education. For example, the group leader read a story, asked interactive questions about the stories and children were required to adopt the mental perspectives of other people within the story. Training was frequently supported by age-appropriate playful components such as hand puppets, drawings and/or funny names of the main characters to ensure compliance. For children, socio-cognitive trainings thus provided opportunity to actively think about specific age-appropriate socio-cognitive tasks to develop awareness of mental states as they start to learn to reflect about other people’s perceptions and thoughts. These highly adapted training environments effectively aid ToM development and likely explain the relatively high effect sizes found in our meta-analysis demonstrating that even “healthy” developing children can benefit from socio-cognitive training. Furthermore, there was a difference between socio-cognitive trainings for children in kindergarten age and children at elementary school: training for younger children included age-appropriate materials such as the use of comic books and puppets, whereas training for children at elementary school did not use these but focused their training on group discussions and short-stories.

In contrast, trainings designed for adolescents and adults do not require development of new socio-cognitive abilities from scratch but rather to apply and adapt already existing skills to new situations. Training in these groups also comprised longer sessions (between 30 and 60 min, as our results indicate) and rather complex tasks were used (e.g., reading the mind in the eyes task or social decision making in complex social dilemma scenarios). Furthermore, training for younger and middle-aged adults frequently comprised of psychoeducation with the focus on understanding, perceiving, managing, and using of socio-cognitive tasks. For example, participants were provided information about different emotions, social situations and learning materials about different mental states with the aim to reflect on their own socio-cognitive abilities. Notably, training approaches for young and middle-aged adults were highly standardized with regard to structure, tasks and materials and surprisingly, individual challenges that participants may face in their everyday life requiring specific behavioural adaptations were not addressed. The overall training duration was rather long, ranging from eight and 39 weeks and training sessions were no longer than 60 min to accommodate work schedules. Results indicate that trainings were effective in improving ToM, emotion recognition, as well as perspective taking in middle-aged adults.

Because socio-cognitive abilities may start to decline with more advanced age^17^, the aim of socio-cognitive training in individuals over the age of around 60 years was to assist maintenance or improvement of these abilities. Training for older adults mainly comprised practicing specific ToM-tasks (e.g., reading a complex false-belief story that includes misunderstandings, double bluffs or sarcasm and conversations about different perspectives in stories) as well as conversations about mental states (e.g., what do the people in the false-belief story think about the situation and about the mental states of the other characters in the story?). Conversations about mental states were described as particularly important in older adults to address lack of social interaction due to reduced social network sizes^50,51^. Surprisingly, none of the reviewed studies in older adults trained specific cognitive processes that are frequently impaired in aging and interact with socio-cognitive processes (e.g. working memory, attention, processing speed^52^). Duration of training sessions was two hours in all investigated studies. A longer duration was chosen to provide older participants with enough time to complete the respective tasks without feeling rushed. Importantly, the meta-analysis suggests that socio-cognitive training was even more effective in healthy older adults than in children. Future research needs to clarify the effective elements in the respective training approaches responsible for this effect (e.g., differences in duration, frequency, content).

Overall, socio-cognitive abilities are multi-componential and involve several processes at a number of levels from cognitive, perceptual to conceptual^53^, which can all be targeted in effective interventions. Overall, different training studies highlight the need to include a variety of training tasks to promote generalization^46^. Given the fact that one of the most desirable outcomes of a specific training are transfer effects to other untrained tasks, research should focus on designing trainings that are able to produce transfer or generalization to several socio-cognitive (sub-) domains^46^, e.g. by incorporating other cognitive domains that are known to support socio-cognitive skills. Furthermore, as most of the included studies in our review focused on ToM-training, a number of socio-cognitive functions such as emotion recognition, (visual) perspective-taking and social decision making are underrepresented, even though all of these domains are essential for social interaction in everyday life^1^. Yet, it is conceivable that training more complex tasks like ToM-exercises and interpretation of complex social situations leads to more pronounced generalization than the training of single socio-cognitive functions. However, direct comparisons of these approaches are currently not available. Still, our review suggests that there is a gap in socio-cognitive training research and training studies focusing on emotion recognition, visual perspective taking, and social decision making are currently lacking.

Several factors influence the development of socio-cognitive abilities during childhood, among them e.g. social factors as family discourse about emotions^54^, number of siblings^55^, and interaction styles among family members^56^. However, cross-cultural studies have demonstrated that these social factors are not identical in different cultures and children may develop socio-cognitive abilities via different pathways (e.g. by talking about other people’s mental states instead of their own mental states) depending on their cultural background^31^. Indeed, except for studies in the upper age-bracket which were all conducted in Italy, the reviewed socio-cognitive studies were conducted in different cultural backgrounds. While the meta-analysis demonstrated that all studies were effective in improving socio-cognitive abilities, it is worth noting that training materials were culturally-sensitive and adapted to the specific culture they were used in (e.g. European/American/Asian faces in Emotion Recognition Tasks). This highlights the utmost importance to not only consider social and aging contexts, but also the cultural aspects in training designs^57^.

Importantly, the results of our meta-analysis showed substantial positive effects of socio-cognitive training on ToM in all investigated age-groups. However, we were not able to consider effects on affective (i.e., inferences regarding others’ emotions) ToM and cognitive ToM (i.e., inferences concerning others’ beliefs and knowledge^58^) separately, because not enough data was provided on these two constructs. However, this would be relevant as cognitive ToM shows greater age-related decline than affective ToM^51^. Table 4 provides an overview of identified areas for future research in the field of social cognitive trainings, as well as general recommendations for training studies. In addition, data long-term maintenance of training effects was rarely reported or comprised different timings of the assessments and could not be considered in the meta-analysis. Also, all our analyses showed a high heterogeneity, which did not decrease in our sensitivity analysis where we divided the studies according to different theory of mind tests used (e.g. false-belief task, strange story test, socio-cognitive composite score), thus limiting our meta-analytic results. Contributors to this unexplained heterogeneity may include variability in socio-cognitive training content and duration (which we therefore described in detail in our included tables), as well as potentially different socio-demographics and personality factors in the studied samples, which may not be discernible from published results. To account for this, future studies may try to conduct an individual patient data meta-analytic approach in which original data from all included studies will be compared and detailed mediation or moderation analysis are possible.

**Table 4:** Recommendations for future studies.

Recommendations for…	
Future studies in the field of socio-cognitive training	*Social cognition is more than just ToM* (future studies may focus on socio-cognitive domains such as emotion recognition training, social decision making, visual perspective training) *Consideration of possible predictors* (future studies should consider and assess possible sociodemographic and/or cultural predictors that may influence training performance) *Assessment of transfer effects* (future studies should assess transfer effects to assess a possible generalization of training effects) *Assessment over the lifespan* (future studies may also focus on socio-cognitive skills and training in middle-aged adults)
Study design and documentation in training studies	*Preregistration* (all future intervention studies should be registered prior to the start of data collection) *Randomization* (participants should be randomly assigned to conditions; randomization requires large sample sizes) *Sampling* (sample sizes should be large enough to observe effects of reasonable size reliably) *Blinding* (blinding should be ensured whenever possible for researchers and participants) *Clear definition of constructs* (future studies should clearly define socio-cognitive domains and subdomains, e.g. affective/cognitive ToM) *Good control conditions* (a baseline control condition, ideally an active control condition should be used as it is more critical for inferences about the potency of the intervention than a passive control group) *Reliable outcome measure* (outcome measures with known or measurable reliability should be used and reported)
Analysis, reporting and publication of training studies	*Correction for multiple comparisons* (corrections for multiple comparisons should be used to adjust the alpha level) *Open data policy* (data and analysis plans should be provided publicly) *Single publication* (duplicate publications should be avoided or described and explained in detail with reference to the original study)

Please also refer to general reporting guidelines and recommendations of cognitive /socio-cognitive training studies, such as e.g.^59,60^, from which we adopted the general recommendations for study design, documentation, analysis, reporting, and publication of training studies.

Transfer effects have only been assessed in few studies. Consequently, more research in these areas is needed. In addition, many studies were not pre-registered and information about participant randomization was frequently lacking, which needs to be addressed in the future to reduce risk of bias.

In sum, effective and adaptive socio-cognitive functioning is crucial throughout the human lifespan and identifying effective training elements may contribute to preventing mental and physical disease and to increase quality of life in various populations. The present systematic review and meta-analysis highlights the specific characteristics of training approaches in different age groups and confirms the effectiveness of these approaches across the healthy human lifespan.

## Methods

The present systematic review and meta-analysis was pre-registered. The review protocol is available at www.crd.york.ac.uk/PROSPERO/ (ID: CRD42020193297). Reporting follows the Preferred Reporting Items for Systematic Reviews and Meta-Analyses (PRISMA) guideline^25^. See Supplementary Material Tables 1 and 2 for the “PRISMA for Abstracts Checklist” and the “PRISMA checklist for systematic reviews”.

### Systematic review

A systematic literature review was conducted to describe and examine the characteristics and effects of social cognition training approaches in healthy participants across the human lifespan, using the highest reporting standards in this field. In the following, the search and study selection processes for the systematic review are described, as well as eligibility criteria for study inclusion, data selection processes and quality assessment procedures.

### Search and study selection

MEDLINE Ovid, Web of Science Core Collection, CENTRAL, and PsycInfo databases were searched for social cognition training studies up to 15th January 2021. An update search was performed up to the 1st September 2021. We also searched for additional studies in reference lists of relevant reviews (e.g. Hofmann et al.^61^). In cases where no full text could be obtained, we contacted the authors and requested full text publications within a 10-day time frame. As an example, the full search string for MEDLINE is described in the Supplementary Materials, Table 3.

### Eligibility criteria

The “Participant, intervention, comparison, outcome” (PICO)-system was used^62^ to define eligibility criteria. Studies were considered eligible if they had included healthy female and male participants of all ages (P). Studies that had included patients with diagnosis of any psychiatric or other medical diseases were excluded. Randomized controlled trials (RCTs) as well as controlled studies investigating interventions focusing on training social-cognitive skills were included in the systematic review (I). No further pre-specifications about details of the interventions were made. Control Groups in eligible studies were required to have received either a different type of socio-cognitive intervention in healthy participants of all ages or participated in passive control and/or waitlist control group (C).

Behavioural changes in ToM tasks were defined as primary outcome (O). ToM is defined as the ability to attribute mental states to others or the ability to understand and predict others’ behaviour based on their mental states and is the most frequently studied socio-cognitive process across development and in healthy and pathological aging^63,64^. Secondary outcomes were chosen to represent three additional major socio-cognitive domains: social perception^1^ (recognizing others as “living persons” via the analysis of perceptual information including e.g. emotion recognition and visual perspective taking^65^), social understanding (using the social perception input for higher-level processes, e.g. theory of mind^66^), and social decision-making (using the obtained social information for social decision making^67^). Studies had to be published in English or German to be included. Only direct pre-post intervention outcome data was considered because the timing of reported long-term effects was too heterogeneous to allow for reasonable comparison between studies. However, Table 2 provides an overview of all assessment time-points for each study, wherever applicable.

### Data extraction

Only studies that fulfilled all of the above-mentioned inclusion criteria were included in the present review. From these studies, data was extracted using a standardized extraction form by two reviewers (MR, KH). If no consensus could be reached, a third author (MM) was contacted for a final decision. Authors of specific studies were contacted for additional information, if required^68^.

### Risk of bias

Risk of Bias was assessed using the Revised Cochrane risk-of-bias tool for randomized trials (RoB2 tool^69^, which implements signalling questions for five domains leading to low/high/medium concern for risk of bias). Two review authors independently assessed risk of bias for each study (MR, KH).

### Meta-analysis

A random-effects pairwise meta-analysis was conducted to calculate the overall effect of socio-cognitive training compared to passive control interventions. Direct comparison of different socio-cognitive trainings was not possible because data was too sparse and heterogeneous to perform a pairwise meta-analysis. Data analysis was conducted using R^70^. For all analyses, the alpha level was set at 0.05. Our primary outcome “theory of mind” was the dependent variable for the meta-analysis. Secondary outcomes could not be considered in the meta-analysis because there was not enough data available. Three broad age-subgroups were defined comprising studies which had included participants between 0 and 18 years, participants between 19 and 59 years, and participants older than 60 years for a first overview and clustering of studies. Yet, in the lowest age bracket, we identified studies in children between 4 and 5 years and 7–9 years old children who have started attending school (i.e., reflecting two major challenges for socio-cognitive development). No studies on socio-cognitive trainings in adolescents were identified. In addition, no studies investigating participants aged between 40 and 60 years could be identified. Consequently, the middle age bracket included only young adults. Only RCTs were considered for the meta-analysis. Data were independently extracted by two reviewers (MR, KH). The mean change from baseline to post-intervention, standard deviation of the mean change, and the number of evaluated participants in each intervention group were used to calculate standardized mean differences. As for the systematic review, only immediate effects were considered because data on long-term effects was limited and heterogeneous timings. We used the I^2^ statistic to address heterogeneity of the included studies. As recommended in the Cochrane Handbook for systematic reviews of interventions^68^, we interpreted heterogeneity as follows: 0–40%: not important/low heterogeneity; 30–60%: moderate heterogeneity; 50–90%: substantial heterogeneity; 75–100%: considerable heterogeneity. A funnel plot for identifying possible publication bias was calculated.

Sensitivity analyses were conducted using fixed effect models. In addition, we further divided our sample according to different theory of mind tests used in the studies to investigate whether different ToM tests (e.g., false-belief task, strange story task) have an influence on the effects. Results of the sensitivity analysis are illustrated in the Supplementary Materials, Fig. 1. The data that support the findings of this study are available from the corresponding author upon request.

## Supplementary Information

Supplementary Information.

## Data Availability

Data available on request from the authors.
